# Extended Follow-Up Following a Phase 2b Randomized Trial of the Candidate Malaria Vaccines FP9 ME-TRAP and MVA ME-TRAP among Children in Kenya

**DOI:** 10.1371/journal.pone.0000707

**Published:** 2007-08-15

**Authors:** Philip Bejon, Edna Ogada, Tabitha Mwangi, Paul Milligan, Trudie Lang, Greg Fegan, Sarah C. Gilbert, Norbert Peshu, Kevin Marsh, Adrian V.S. Hill

**Affiliations:** 1 Kenya Medical Research Institute, Centre for Geographical Medicine Research (Coast), Kilifi, Kenya; 2 Centre for Clinical Vaccinology and Tropical Medicine, University of Oxford, Oxford, United Kingdom; 3 London School of Hygiene and Tropical Medicine, London, United Kingdom; 4 Wellcome Trust Centre for Human Genetics, University of Oxford, Oxford, United Kingdom; 5 Nuffield Department of Clinical Medicine, Oxford University, John Radcliffe Hospital, Oxford, United Kingdom; London School of Hygiene & Tropical Medicine, United Kingdom

## Abstract

**Background:**

“FFM ME-TRAP” is sequential immunisation with two attenuated poxvirus vectors (FP9 and modified vaccinia virus Ankara) delivering the pre-erythrocytic malaria antigen ME-TRAP. Over nine months follow-up in our original study, there was no evidence that FFM ME-TRAP provided protection against malaria. The incidence of malaria was slightly higher in children who received FFM ME-TRAP, but this was not statistically significant (hazard ratio 1.5, 95% CI 1.0-2.3). Although the study was unblinded, another nine months follow-up was planned to monitor the incidence of malaria and other serious adverse events.

**Methods and Findings:**

405 children aged 1–6 yrs were initially randomized to vaccination with either FFM ME-TRAP or control (rabies vaccine). 380 children were still available for follow-up after the first nine months. Children were seen weekly and whenever they were unwell for nine months monitoring. The axillary temperature was measured, and blood films taken when febrile. The primary analysis was time to parasitaemia >2,500/µl. During the second nine months monitoring, 49 events met the primary endpoint (febrile malaria with parasites >2,500/µl) in the Intention To Treat (ITT) group. 23 events occurred among the 189 children in the FFM ME-TRAP group, and 26 among the 194 children in the control group. In the full 18 months of monitoring, there were 63 events in the FFM ME-TRAP group and 60 in the control group (HR = 1.2, CI 0.84-1.73, p = 0.35). There was no evidence that the HR changed over the 18 months (test for interaction between time and vaccination p = 0.11).

**Conclusions:**

Vaccination with FFM ME-TRAP was not protective against malaria in this study. Malaria incidence during 18 months of surveillance was similar in both vaccine groups.

**Trial Registration:**

Controlled-Trials.com ISRCTN88335123

## Introduction

There were 515 million episodes of clinical *P. falciparum* malaria in 2002 [1]. Prime boost vaccination with FP9 (an attenuated fowlpox virus) then modified vaccinia virus Ankara (MVA), both recombinant for the pre-erythrocytic antigen construct ME-TRAP (the Multiple Epitope string and Thrombospondin Related Adhesion Protein, ME- TRAP [2]) is safe, immunogenic and partially protective in malaria-naïve adults exposed to experimental challenge [3]. We previously conducted a Phase 2b study of the FFM ME-TRAP regimen (i.e. two sequential FP9 ME-TRAP vaccinations followed by MVA ME-TRAP) to assess efficacy in children living in rural Kenya, and have reported the primary analysis [4]. Local and systemic reactogenicity was mild. Immunogenicity was lower than that seen among partially protected volunteers in sporozoite challenge studies [3] and earlier phase 1 studies of children at lower malaria transmission intensities [5]. There was no evidence of protection against malaria, (the incidence was higher among children vaccinated with FFM ME-TRAP, but this difference was not significant (the hazard ratio was 1.5, 95% CI 1.0-2.3, P = 0.14) [4].

Although the study was unblinded at 9 months for the primary analysis of efficacy, the analysis plan specified continued surveillance for serious adverse events and malaria for a further period of 9 months.

## Methods

The protocols for this trial and supporting CONSORT checklist are available as supporting information; see Checklist S1, Protocols S1, S2, S3, and Analysis Plan S1.

### Study Design

The study was randomised, controlled and double blind. Details of ethical approval are described previously [4].

405 children were randomized, and all received at least one dose of vaccine. Children were screened in February 2005, immunised between March 2005 and May 2005, and followed up until February 2006, when the study was unblinded. A further 9 months follow up was conducted on 387 children who were still available, until November 2006. No further cross-sectional bleeds were taken during this time.

### Participants

The participating children were aged 1–6 years old when randomized in February 2005, healthy, and resident in the study area.

### Location

The study was carried out in Junju sublocation in Kilifi District, on the Kenyan coast, as described previously [4].

### Interventions

The trial vaccination regimen was a candidate prime boost malaria vaccination. Details have been described previously [4]. Briefly, two sequential FP9 ME-TRAP vaccinations (5×10^7^ plaque forming units) followed by MVA ME-TRAP vaccination (1.5×10^8^ plaque forming units), given intradermally. The control was rabies vaccine (Aventis Pasteur, WISTAR strain), administered according to the same timings. Rabies was also given intradermally, at 0.25 IU. Vaccinations were spaced 4 weeks apart (acceptable range 3–5 weeks).

### Objectives

The objectives of this extension of the original Phase 2b trial were to describe the distribution of febrile malaria by vaccination group, and to assess any latent safety issues.

### Outcomes

Efficacy (Malaria episodes)

The primary endpoint was a clinical episode of malaria, defined as an axillary temperature greater than 37.5 degrees centigrade, with a *Plasmodium falciparum* parasitaemia greater than 2,500 parasites per ul. Episodes of malaria were identified as described previously [4].

### Randomization

Full details of randomization have been described previously [4]. Briefly, the investigators in Kenya enrolled children, and applied study numbers sequentially. A list of eligible children was ordered according to age and village, and matched to the list of randomization card numbers, generated in the UK.

### Blinding

The nurses who administered vaccinations did not take part in any other trial related procedure, and were subsequently based in Kilifi District Hospital rather than the trial site. They drew up vaccinations according to the instructions in the randomisation envelope, and documented the vaccination in notes that were not available to the investigators until after unblinding

After the first 9 months monitoring the primary analysis was conducted. A cleaned, locked database was transmitted to the DSMB, and the statistician then transmitted the allocation code to the lead investigator, who then implemented the analysis plan. It was planned to conduct a further analysis of episodes of malaria after a further 9 months monitoring, despite having unblinded the trial. The results (no significant efficacy) were fed back to the study participants, and a strong view was expressed that study participants wished to be told which vaccines children had received. This was done. Continued monitoring was supervised by a second investigator, and the lead investigator played no further part in assessment of malaria episodes or collection of data. The second investigator did not have details of vaccination allocation, neither did the laboratory staff who read blood films. Data was cleaned by the second investigator before being locked, following which analysis was conducted by the lead investigator.

### Statistical methods

The analysis plan was approved by the DSMB. The primary analysis was a log rank test comparing the time to the first or only episode of malaria (defined as fever with parasitaemia above 2500/µL) between the vaccination groups, stratified by age group, ITN (insecticide treated net) use and village, analysed by Intention To Treat. The hazard ratio and 95% confidence interval was estimated by Cox's regression adjusted for the same covariates. Age group was a categorical variable with three levels (1–2 years old, 2–5 years old, 5–6 years old). Village had 5 levels. ITN use was defined as sleeping under a treated net every night, which had less than three holes into which a finger could comfortably fit.

Poisson regression was used to estimate the incidence rate ratio taking into account all malaria episodes, adjusted for the same covariates. A period of 28 days after each malaria episode was deducted from the person time at risk, since individuals were assumed not to be at risk of malaria during this period. No further analysis is planned.

In order to assess interactions, data from each individual was split according to monitoring period. An interaction between vaccination and time of monitoring (first 9 months vs second 9 months) was then assessed for poisson regression and for survival analysis. Models with this interaction were compared with models without an interaction term using the likelihood ratio test.

## Results

### Participant flow

**Figure 1:**
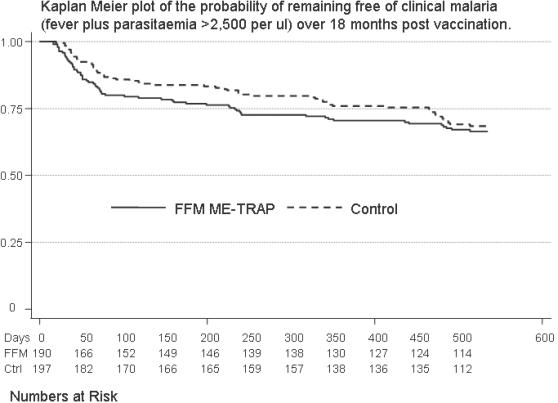
Trial Profile. After screening for eligibility, parents were invited to bring their children back to the dispensary for immunisation. Children were randomized on attending for vaccination. Of the 17 children who attended for the first, but not the final, vaccination, two had moved out of the area, and parents of the remaining 15 chose not to reattend. No severe adverse events were identified in this children. 8 children moved out of the area before 9 months monitoring was complete.

405 children were randomized in May 2005, and 383 were still available for follow up after the first 9 months (Figure 1). 333 completed the second 9 months follow up.

### Baseline data

The treatment allocation groups at the start of the second 9 months monitoring were still well balanced with respect to baseline characteristics (Table 1).

**Table 1:** Covariates by vaccination group at the beginning of the second 9 months monitoring.

	FFM ME-TRAP	Rabies
Village		
Gongoni	36	40
Junju	50	50
Kolewa	49	56
Mapawa	39	32
Mwembe tsungu	15	16
Age Cat (years)		
1–2	36	41
2–5	106	106
5–7	47	47
Bednet*		
Without	75	70
With	113	123

### Numbers analyzed

Of the 383 children followed up in the second 9 months, 342 were from the original According To Protocol analysis group. All 383 were part of the Intention To Treat group.

### Outcomes and estimation

**Figure 2:**
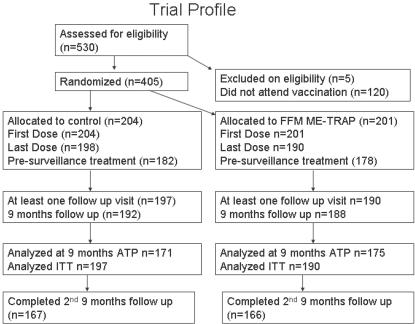
Primary analysis of efficacy. The probability of remaining free of clinical malaria is plotted over the 18 months of monitoring (ITT analysis). Numbers of children at risk are given below the Kaplan Meier plot. The endpoint is >2,500 parasites per microlitre and fever. HR = 0.84, 95% CI 0.47-1.49, p = 0.59

The primary analysis for this study has already been conducted [4]. During the second 9 months monitoring, there were 49 events that met the primary endpoint (febrile malaria with parasites >2,500/µl) in the ITT group. 23 of these occurred among the 189 children in the FFM ME-TRAP group, and 26 among the 194 children in the rabies control group (logrank test p = 0.59, p = 0.67 when stratified by age, village and ITN use). In the full 18 months of monitoring, there were 63 events in the FFM ME-TRAP group and 60 in the control group (p = 0.46, p = 0.35 when stratified by covariates). A Kaplan Meier plot of the ITT group is shown in figure 2.

When analysis was restricted According To Protocol, there were 52 events among 170 children in the FFM ME-TRAP group, compared with 54 among 172 children in the rabies control group (p = 0.94, p = 0.95 adjusted).

### Survival Analysis (Cox Regression)

The hazard ratio (HR) for the effect of vaccination, estimated using Cox regression, was 1.2 (95% CI 0.84-1.73, p = 0.35) over the full 18 months of monitoring. The HR during the first 9 months was 1.52 (1–2.31, p = 0.14), followed by 0.84 (0.47–1.49, p = 0.59) during the second 9 months. When analysis was restricted to ATP, the HRs were 1.06 (0.72–1.56 p = 0.94), 1.31 (0.83–2.08, p = 0.35) and 0.72 (0.39–1.34, p = 0.31) for the full 18 months, first 9 months and second 9 months monitoring, respectively. The difference in HRs between the two time periods was not statistically significant (the likelihood ratio test for the interaction between time period and vaccination group gave p = 0.11 and p = 0.15 for ITT and ATP analyses respectively).

### Multiple Episodes

During 18 months monitoring of the ITT cohort there were 170 episodes of febrile malaria with parasitaemia >2,500/µl among 383 children. This comprised 0.35 episodes per Person Year At Risk among children given FFM ME-TRAP, and 0.29 among children given the control vaccination.

The Incidence Rate Ratios for the effect of vaccination were 1.06 (CI 0.76-1.48, p = 0.75) during the first 18 months, 1.58 (CI 1.08-2.32, p = 0.017) during the first 9 months and 0.8 (0.46–1.36, p = 0.42) during the second 9 months. Among the ATP cohort the IRRs were 1.06 (0.76–1.48, p = 0.75), 1.36 (0.89–2.06, p = 0.16) and 0.67 (0.38–1.19, p = 0.17) for the full 18 months, first 9 months and second 9 months monitoring, respectively. The likelihood ratio test for the interaction between vaccine group and time period gave p = 0.051 and p = 0.093 for ITT and ATP analysis respectively.

### Adverse Events

8 Further serious adverse events were detected during the second 9 months of follow up. Among FFM ME-TRAP vaccinees there were episodes of pneumonia, dysentery, malaria with multiple seizures and a snake bite which required hospital admissions. Among control vaccinees there were two episodes of gastroenteritis with dehydration, an episode of malaria with multiple seizures and a Stevens Johnson reaction (associated with co-trimoxazole use). All adverse events resolved without sequelae, and there were no deaths in either group.

## Discussion

### Interpretation

We have previously reported the primary analysis after 9 months post-vaccination monitoring for episodes of malaria [4], during which there was no evidence of protection against malaria. The incidence was higher among children vaccinated with FFM ME-TRAP, but this difference was not significant. We report here a further 9 months monitoring of the same children, during which the incidence of malaria episodes was similar in both groups.

The primary analysis considers time to first episode. In this analysis children do not contribute further to the analysis after an episode of malaria, which could potentially cause a bias in the estimation of the hazard ratio in the second period. However, similar results were obtained when multiple episodes were analyzed.

The primary analysis had already been conducted. The analysis here is therefore considered secondary. Furthermore, these data were acquired after the study had been unblinded. Measures were taken to reduce the impact of this on the integrity of the study; an investigator not involved in the primary analysis oversaw the second 9 months of monitoring, and blood films were read in the laboratory with reference to only study subject numbers without knowledge of vaccination group. The lead investigator who had conducted the primary analysis was not involved in continued follow up, and conducted the analysis presented here after being sent a cleaned database. That no efficacy was seen on the primary analysis was communicated to study participants, and so vaccine allocation was perhaps unlikely to bias health seeking behaviour. A similar number of follow up visits were completed among children allocated to either vaccination (4,958 visits among FFM ME-TRAP vaccinees vs 5,130 among controls), and a similar number presented for assessment of fever between visits (1,086 among FFM ME-TRAP vaccinees vs 1,095 among controls), p = 0.58). Nevertheless, a subtle impact on health seeking behaviour cannot be excluded, and our findings here must be treated with appropriate caution.

Borderline significance on log likelihood testing for interactions between monitoring period and vaccination was seen. However, this was one of multiple analyses for interactions. The other 3 tests yielded less significant findings, and the study was no longer blind. There is insufficient evidence to conclude there might have been an interaction between vaccination and time (i.e. the effect, or lack of effect, of vaccination was constant throughout the duration of the study). This suggests that the variations in hazard during the monitoring period probably represent chance alone.

After the primary analysis of this trial we considered the formal possibility that transforming growth factor β production [6] and anti-inflammatory responses [7] induced by vaccination might increase susceptibility to malaria. However, there was no indication that higher T cell responses predicted greater susceptibility to malaria, suggesting that there was not a causal link, and the increase in risk was not significant during primary analysis [4]. The convergence of Incidence Rates and Hazards seen during the second 9 months of monitoring provides further reassurance. Since the vaccinated children are now aged 3–8 years, and the incidence of malaria is expected to be lower still, continued analysis of episodes of malaria by vaccination group is not planned.

## Supporting Information

Protocol S1 Protocol Under Which Further 9 Months Follow up was Conducted
(4.42 MB PDF)

Protocol S2 Protocol for ongoing study
(4.42 MB DOC)

Protocol S3 Original protocol
(0.31 MB DOC)

Analysis Plan S1 Analysis Plan for second 9 months follow up
(0.03 MB DOC)

Checklist S1 CONSORT checklist
(0.05 MB DOC)
