# Supplementary material for: Extended Follow-Up Following a Phase 2b Randomized Trial of the Candidate Malaria Vaccines FP9 ME-TRAP and MVA ME-TRAP among Children in Kenya
Source: PLoS One. 2007 Aug 15;2(8):e707. doi: 10.1371/journal.pone.0000707 (PMC1940326; doi:10.1371/journal.pone.0000707)
Supplement: Protocol S1 — Protocol Under Which Further 9 Months Follow up was Conducted (4.42 MB PDF) [file pone.0000707.s001.pdf]

1. **Title of the Project:** **Integrated studies of the development of natural immunity to malaria in children in Kilifi district.**

2. **Investigators and Institutional Affiliations**:


K Marsh KEMRI CGMRC (Principal investigator)

T Williams KEMRI CGMRC

T Mwangi KEMRI CGMRC

S Kinyanjui KEMRI CGMRC

B Lowe KEMRI CGMRC

F Ndungu KEMRI CGMRC

P Bejon KEMRI CGMRC

P Bull KEMRI CGMRC

F Osier KEMRI CGMRC

C Mugenyhi KEMRI CGMRC

G Fegan KEMRI CGMRC

G Warimwe KEMRI CGMRC

E nduati KEMRI CGMRC

L Ochola KEMRI CGMR

B Urban, KEMRI CGMRC

E Ogada KEMRI CGMRC

M Mackinnon KEMRI CGMRC

D Conway London School of Hygiene and Tropical Medicine

J Langhorne. MRC Mill Hill London

J Beeson Walter and Eliza hall Institute Melbourne,

A Farnert .Karolinska Institute Stockholm

## ABBREVIATIONS

AMA Apical membrane antigen

DBL Duffy binding like domain

CD Cluster of differentiation (cell surface typing system identified by number eg CD36

CR1 Complement receptor 1

ICAM1 Intra cellular adhesion molecule 1

FACS Fluorescence activated cell sorter

IRBC Infected red blood cell surface

KDH Kilifi District Hospital

MSP Merozoite surface protein

PCR Polymerase chain reaction

WEHI Walter and Eliza hall Institute


3. **Abstract**:

Malaria is one of the major health problems facing Kenya and the rest of sub Saharan Africa. Current preventative and treatment approaches are at best holding strategies, continuously compromised by problems of drug resistance, costs and logistic problems. The main hope for fundamentally changing this situation is the development of malaria vaccines. An important part of this process is the understanding of the mechanisms of naturally acquired immunity to malaria in people living in malaria endemic areas. A key requirement for longitudinal studies of putative protective responses is the ability to define immune status. Using longitudinal measurement of clinical disease has become a standard approach to do this. At the KEMRI Centre for Geographic Medicine Research Kilifi (CGMRC) we have previously established cohorts in which we have derived local definitions of clinical malaria. We have also established surveillance systems for the detection and definition of cases of severe malaria admitted to hospital Here we propose the continued monitoring of two community based cohorts over the next four years and the recruitment of cases of severe disease and non severe disease at health facilities. Using this sampling framework we will investigate the relationship between specific immune responses and protection from clinical malaria.

## 4. Introduction/Background:

Childhood mortality due to malaria has risen over the last twenty years[1]. It is one of the few diseases where the introduction of an effective vaccine can be said to be genuinely essential to achieve a decisive change. This being the case, a detailed understanding of the mechanisms of human immunity to malaria is important both in identifying targets and mechanisms and in monitoring human responses to candidate vaccines.

This proposal seeks to build on and maintain the epidemiological infrastructure that we have established at Kilifi and to carry out an integrated programme of work aimed at achieving a comprehensive picture of the targets and mechanisms of human immunity to *P. falciparum* malaria.

### *4.1 Immunity to malaria and the design of field studies*

That humans living in endemic areas develop immunity to malaria is evident from the fact that in such areas the burden of disease falls on young children. Older children and adults are resistant to severe morbidity and death, though remaining susceptible to infection[2]. Despite a large number of field studies over many years, knowledge on the key targets and mechanisms of blood stage immunity is surprisingly limited, with many studies giving negative or contradictory results. However recent advances in understanding the epidemiological picture of immunity, coupled with new techniques in molecular parasitology, present an opportunity to now develop a better understanding. We propose that there are three key issues which need to be tackled in order to establish a clear picture of the mechanisms of human immunity to malaria (1) The definition of functional protective status of individuals (ie who is immune, and what are they protected from?). (2) The definition of immune responsiveness of individuals. (3) The need to adequately take account of antigenic polymorphism and antigenic variation of the infecting isolate.

#### *4.1.1 Who is immune, and to what?*

To test the hypothesis that a candidate immune response is important in protection from malaria requires that one is able to compare susceptibility in individuals with and without the response. The patterns of three indicators of human immunity to malaria in endemic areas: parasite prevalence, period prevalence of clinical attacks and period prevalence of severe life threatening attacks all show evidence of acquisition of resistance with increasing age but the indicators have quite different kinetics [2,13]. From the point of view of both vaccines and of the evolution of the human immune respons[e], protection against severe, life threatening, disease is fundamentally important. We have previously shown that significant immunity to severe disease is acquired after relatively few symptomatic infections[3]. Immunity to life threatening disease is established at a time when susceptibility to ordinary episodes of clinical malaria changes little and therefore protection from symptomatic malaria cannot be assumed to measure surrogates of protection from severe disease. Thus it is essential to define an individuals status in terms of susceptibility over time to clearly defined levels of disease, and ideally to examine outcome in relation to severe disease. Over the last 15 years we have characterised in detail severe malaria in African children[4], and more recently we have derived case definitions for mild disease at different ages and under different transmission conditions, optimised for sensitivity and specificity[5].

#### *4.1.2 Defining immune responsiveness*

Once issues concerned with definitions of protective status have been addressed, the standard approach is to measure a candidate immune response and relate it to incidence over time of clinical

episodes of disease. However, this assumes that a meaningful estimate of the individual's capacity to mount the response in question can be made at a single time point. Over the last few years we have made a series of observations which suggest that this is not the case and that misclassification of immune responsiveness may potentially confound the interpretation of field studies of immunity[6,7]. In these studies we found that the amount of antibody in individuals who were parasitised at the time of sampling is greater than in those who were parasite negative. Given that all children in this age range show evidence of exposure, and that the move between being parasite positive and parasite negative occurs frequently, the implication is that the ability to detect a given immune responses may be short lasting. We have subsequently confirmed this in studies of the kinetics of a number of specific responses[8]. In individuals who are parasite negative at the time of sampling it may therefore be impossible to distinguish those who genuinely have not made a particular response from those who would be capable of making it if challenged, resulting in misclassification errors. The implications of this phenomena are clear in several of our recent studies where analysis of antibody responses for the population as a whole to a range of antigens revealed no protective effect, whereas in the individuals who were parasite positive at the time of sampling a clear protective effect was evident [6,7]. Thus it is essential to examine immune responsiveness in individuals over time and to conduct studies large enough to allow account to be taken of potential misclassification.

#### *4.1.3 Accounting for antigenic polymorphism and antigenic variation.*

Observations made during the use of malaria therapy for the treatment of neurosyphillis indicated that an important component of immunity to malaria is "strain" specific [9]. Parasite heterogeneity and specificity of the resulting immune response is a plausible explanation of the need for prolonged exposure to achieve immunity to clinical episodes of malaria [10]. Putative target antigens for protective immune responses show considerable polymorphism either through the existence of alternative allelic forms in the case of many key merozoite antigens[11] or through clonal antigenic variation in the case of important targets expressed on the infected red cell surface[12]. The emerging evidence of protective responses being directed to such polymorphic and variant epitopes implies that it is unlikely that immune status will ever be defined by any single response. Rather, it is likely that in different individuals different responses will be more or less important and that any individual will rely on several different responses, which are likely to be additive or synergistic. This has implications for the design of studies, as the maximum contribution to overall protection from a single response, no matter how effective, to a single polymorphic form of a single antigen is likely to be small. However, the majority of field studies carried out to date have examined a very limited range of responses, often not taking account of known polymorphisms and usually not relating polymorphic responses to the phenotype or genotype of infecting parasites. Thus it is essential in longitudinal studies to relate putative protective response to the phenotype or genotype of the parasites causing disease and to examine multiple responses within the same epidemiological framework.

### *4. 2 Key areas of research*

Protective immune responses to the malaria parasite could be directed at either the sporozoite, the hepatic stages, free merozoites as they attempt to invade the host red blood cell, or at the infected red cell containing the maturing parasite. Not surprisingly, the molecules and processes that form potential targets for immune responses are also potentially important in parasite virulence. Once targets for immune responses are identified it is important to understand how host parasite interactions lead to regulation of the immune response. Because this proposal focuses on establishing and maintaining the epidemiological framework for examining responses to all stages of the parasite we have concentrated on the epidemiological aspects of the design. A concise summary

of the technical background to immunity to these stages is contained in reference 13. Details of proposed studies on potentially protective mechanisms and antigens are summarised in the methods section of the proposal.

### *4.2.1 Epidemiological framework*

Longitudinal studies, with careful documentation of disease episodes, remain the standard approach to identifying potential protective immune responses. Although this approach *on its own* could potentially miss mechanisms specific for protection against severe disease (as discussed above), the converse is unlikely to be true (ie any response that reduces the risk of clinical episodes of non severe disease is likely to also protect against more severe disease). Cohort studies of non severe disease, which by their nature require a much smaller number of subjects to be followed, allow more detailed observations to be made and provide a key screen for rapid identification of potentially important immune responses. In 1998 we initiated two cohort studies in areas within 25km of each other in Kilifi District, involving populations of similar ethnic composition and socio-economic status but differing in malaria transmission (SSC numbers 485 and 669) The current proposal will replace and supersede procedures set up under these two protocols. Ngerenya to the north of Kilifi town experiences between 1 and 10 infected bites per year, Chonyi to the south of Kilifi town experiences between 50 and 100 infected bites per year. Between 1998-2001 the main emphasis of work was in establishing age and transmission appropriate definitions of clinical malaria with the optimum balance of specificity and sensitivity[5]. In 2001 follow up of the Chonyi cohort was discontinued in order to concentrate resources on the Ngerenya cohort, which has now been under active surveillance for nearly seven years. Although initially a cohort initiated from a cross section of the population, in each year all births in the study households have been recruited into the cohort, thus over time establishing a birth cohort. This design allows analysis of how immune responses over time relate to an individual's experience of both parasitisation and clinical disease. In addition, interactions between immune responses and modifying factors such as nutritional status[14] and human genetic polymorphisms have also been studied.

As we have subsequently investigated a range of immune responses it has become clear that in many ways the information from the high transmission cohort has been more informative than that from the low transmission area. This is likely to because the sampling from the Chonyi cohort crosses the age range in which there is a marked drop in the incidence of clinical disease (ie the period in which immunity to clinical disease is becoming established), whereas in the Ngerenya cohort this occurs at a later time point We therefore propose to re establish a cohort in the high transmission area whilst continuing to maintain the Ngerenya cohort under observation in order to capture the point individuals enter the window in which disease incidence falls.

### *4.2.2 Protection against severe malaria*

The occurrence of severe malaria provides an unequivocal definition of significant lack of immunity. Furthermore, as discussed above, the kinetics of immunity to severe malaria are distinct and may involve different mechanisms to those protecting from less severe disease. The main reason that this approach is generally not taken in immuno-epidemiological studies is the logistic complexity: severe malaria is a relatively rare event, with a community-based incidence of between 18-40 per 1000 per year[15], requiring very large denominator populations and close integration between an epidemiological framework and a clinical surveillance system. At Kilifi we currently maintain

surveillance of a population of 240,000, representing 80% of the drainage population to Kilifi District hospital (KDH). We also maintain a linked continuous clinical surveillance of all paediatric admissions to the hospital (approximately 5000 per year). In 2003 we initiated recruitment of a birth cohort from within the population under surveillance, which will eventually comprise approximately 5000 individuals (SSC number 613: a cohort study of susceptibility of invasive pneumococcal disease among children aged 0-23 months). All children from within the cohort who present to KDH are identified and detailed investigations carried out to establish an accurate diagnosis and clinical phenotype. This framework allows the identification of cases of malaria of defined severity and of control children matched as necessary (for instance by age, location, hospital utilisation or season). We propose to take a generic approach of performing nested case control analyses to examine the role of defined immune responses in relation to the chance of developing severe malaria. Initial analyses will compare the prevalence and degree of candidate responses at the time point immediately prior to admission, however the longitudinal nature of the design allows further analysis of the development of responses prior to that time, for instance taking account of fluctuations in antibody levels over time which may not be detected in a single time point.

#### *4.2.3 Examining the acute and convalescent response of children with malaria of differing degrees of severity.*

It has generally been assumed that meaningful information on protective immune responses cannot be obtained at the time of acute disease due to the widespread perturbation of immune responses and the inability to relate responses to the status of the individual before challenge. However, from the discussion in section 4.1.2 it is evident that the ability of an individual to mount a particular response may *only* become apparent in the presence of parasites i.e. on challenge. We therefore hypothesise that the differences between evolving immune responses in the acute and immediate convalescent phases in children with severe malaria (ie *inadequate* control of recent challenge) compared with those with mild disease (*better* control of recent challenge) may be critical in identifying important protective responses. We will examine immune responses at the time of acute disease and over the ensuing weeks following treatment in children admitted to hospital with defined clinical phenotypes to KDH, to Malindi District hospital and to outpatient clinics. This approach is complementary to the prospective cohort studies described above and has the additional advantage that one can also study cellular responses potentially involved in immune regulation. The latter is difficult to incorporate into prospective studies because of small sample volumes involved and the potential difficulties of working with cryopreserved cells.

## 5 Justification for the Study:

Malaria is a major cause of childhood mortality and morbidity in Kenya and the rest of Africa. Currently available methods for prevention and treatment face multiple problems including the costs and logistic difficulties of ensuring coverage, in the case of bed nets, and rapidly developing resistance in the case of antimalarial drugs. There is a major international drive to develop anti-malarial vaccines. Understanding the mechanisms by which antimalarial immunity is acquired, and the factors that modify it, are an essential part of efforts to develop a malaria vaccine.

## 6 Null Hypothesis:

This protocol seeks to maintain an established a framework in which to carry out longitudinal studies of immune responses to malaria. As such there is no single null hypothesis; for each immune response of interest the null hypothesis is that the immune mechanism under examination provides no protection against clinical episodes of malaria.

## 7 (a) General Objectives:

To establish an epidemiological framework in which to carry out longitudinal studies of the development of immune responses to malaria

## 7 (b) Specific Objectives

1 To perform active and passive surveillance to detect cases of clinical malaria in two cohorts of children living in a malaria endemic area.

2 To detect episodes of mild and severe malaria in order to compare their prior and evolving immune response.

3 To examine the relationship between defined measures of the immune response to key immunological targets on the sporozoite, merozoite and infected red cell surface in protecting from malaria.

4 To examine the relationship between defined measures of the immune response to key immunological targets on the sporozoite, merozoite and infected red cell surface and key modifying factors such as host genetic polymorphisms, nutritional status and environmental factors.

## 8 Design and Methodology:

Study site and study populations

We will recruit subjects from four separate groups:

**Cohort studies with active case detection:**

These studies will be carried out in Ngerenya and Junju locations in Kilifi district. Ngerenya is an area of low-moderate malaria transmission and Junju is in an area of higher transmission. Both areas have been extensively characterized in previous studies (SSC protocol 485, 669) and 915).

**Detection of cases of severe malaria in an established birth cohort**
In 2003 we initiated recruitment of a birth cohort from within a population under surveillance in Kilifi district, which will eventually comprise approximately 5000 individuals (SSC number 613: a cohort study of susceptibility of invasive pneumococcal disease among children aged 0-23

months). In brief children are seen at twelve-week intervals at which point details of present and past health events are recorded, a physical examination made and a blood sample collected for storage of serum and preparation of a blood film. All children from within the cohort who present to KDH are identified and detailed investigations carried out to establish an accurate diagnosis and clinical phenotype. We will identify all admissions due to malaria (see study procedures below for definition of malaria). Active follow up of the cohort was originally scheduled to end as each child reached two years of age. We now propose to continue limited follow up with twice yearly contact with cohort children until the age of six years.

**Detection of cases of severe malaria outside of an established birth cohort**

The KEMRI centre in Kilifi in collaboration with MOH staff at Kilifi hospital maintain continuous surveillance of all admissions (5000 per year) at Kilifi district hospital. A limited surveillance based on the methods used at Kilifi will also be established at Malindi hospital. Children presenting with severe malaria at both hospitals will be eligible for inclusion to studies to examine the evolving immune response to malaria.

**Recruitment of cases of non severe malaria.**

Children with non severe malaria will be recruited from those attending outpatient clinics at Kilifi District hospital, and at Ministry of health dispensaries at Pingilikani, Junju and Ngerenya.

8.2 Criteria for inclusion of subjects.

**Longitudinal cohort studies in Junju and Ngerenya:** All children and a proportion of adults in Ngerenya living in households previously mapped registered for longitudinal surveillance will be invited to be in this study. New births in these households will be recruited. Children will leave the study when they reach their eleventh birthday. A similar procedure will be used in Junju where households will be recruited from a previously established household register.

**Severe malaria cases at Kilifi and Malindi hospital:** All children admitted to hospital with a history of fever, no other obvious diagnosis at admission and a positive blood film for *P falciparum* asexual stages will be recruited as "potentially severe malaria". Subsequently cases will be dropped if later investigations (eg positive blood culture or lumbar puncture) suggest an alternative primary diagnosis. During analysis different levels of severity will be defined in relation to the presence of signs previously shown to have prognostic significance in Kilifi ie the presence of prostration (including coma) and/or respiratory distress.

**Non severe malaria presenting at outpatients:** Children presenting with a history of fever in whom no other obvious primary cause is detected on examination and who have a blood film positive for asexual stages of *P falciparum* in the case of children under one year of age, or equal to or greater than 2500 per micro litre in children aged over 1 year (these definitions have previously been shown to have the best combination of sensitivity and specificity for defining a case of clinical malaria in Kilifi district).

8.3 Criteria for exclusion of subjects.

Failure of parents to give informed consent or withdrawal of consent at any time during the study.

## 8.4 Sample size determination.

The aim of the cohort studies is to examine a range of potentially protective immune responses requiring different sample sizes. The original cohorts in high and low transmission areas were constructed pragmatically to allow a description of the pattern of clinical malaria with age. A number of different immune responses will be studied which can be expected to have different prevalences and for which it is not possible in advance to know what degree of protection any given immune response will confer. However the studies we have conducted to date give a basis for estimating the potential power of any given cohort size. Immune responses of interest tend to be present in between 30 to 60% of children. Given that Children in Ngerenya have an overall annual incidence of 1 episode of malaria per child per year, assuming the prevalence of a protective immune response was 30% (and not transient), following 400 children for 2 years would allow us to detect a reduction in the incidence of malaria episodes of around 20% with 90% power at the 5% significance level. Assuming 20% missing prior samples (due to temporary migration etc) for any single analysis, we will recruit 500 children to each cohort.

Similarly, the detection of cases of clinical malaria of different severity is intended to provide a framework in which a number of different questions can be tackled. Using a case control approach to examining the differences between responses in children with severe and non severe malaria, and anticipating cumulative seroprevalences of responses to different epitopes of interest of 20-30%, detection of factors (eg absence of antibody) which confer an odds ratio of 2 or more with 90% power will require around 350 cases of severe disease ( using between 1 and 3 controls per case depending on seroprevalence). For instance, based on preliminary genotyping the following case numbers will be available for genotype specific analysis (assuming approx 50% mixed genotypes and the ability to allocate a dominant genotype in half of the mixed cases) : MSP1 Block2: K1 (130), RO33 (95), MAD (53), MSP2 : GroupA (153), Group B (102). This will allow the detection of odds ratios of 3 or more with 90% power for the majority of analyses. It is not possible *a priori* to know what level of protection may be conferred by the presence of a given antibody specificity but for comparison previous studies in Kilifi of responses to highly polymorphic antigens on the infected red cell surface demonstrated significantly reduced risk associated with the presence of homologous responses using 65 cases of disease.[16]

## 8.4 Procedures

Longitudinal cohorts

1000 children in total will be recruited, 500 from Ngerenya and 500 from Junju. Fieldworkers living within the community will visit each child weekly at their homes, interview them and take an axillary temperature. If a child has a temperature ≥37.5°C, a finger prick sample of blood will be taken for a rapid test (Opti-MAL®) for malaria, a blood smear and microtainer sample for subsequent serology and parasite genotyping. The rapid malaria test result will be immediately used to treat the study participant (treatments with Co-artem®).. The blood slide will be taken to the laboratories in KDH and if the slide is positive and the rapid test negative, the information will be relayed to the field-worker and the child started on anti-malarial treatment.

If the parent/guardian of the child reports that the child has been feverish but temperature is <37.5°C, then the fieldworker will be required to visit that child at least 2 times in the next 24 hours, taking an axillary temperature at each visit. If at any point, the temperature is ≥37.5°C, then a rapid test, blood slide and blood sample into a micro tainer will be taken and treatments provided as above if the rapid test is positive. Any child who is unwell but has no malaria nor fever, will be referred to the local dispensary: Gongoni in Junju or Ngerenya dispensary (a KEMRI employed nurse or clinical officer will work in collaboration with the responsible MOH nurse to strengthen clinical services and ensure detection and assesment of cohort children attending the dispensary) . Over the course of the four year surveillance we will examine acute and convalescent responses to acute episodes of malaria in a sub group of patients presenting with clinical malaria. In each location 50 adults will be recruited randomly from study households. Adults will not be followed by routine surveillance but will have blood samples collected at cross sectional surveys (see below).

*Blood sampling in Junju and Ngerenya cohorts*

In addition to the diagnostic blood sampling described above, blood samples will also be taken for two other purposes:

a) During the first year of the study four cross sectional surveys will be performed at three monthly intervals. At three of these surveys finger prick blood samples will be collected into microtainer tubes. At one of the surveys a venous sample of blood (maximum 5 mls) will be taken. This will be used to prepare blood films, measure full blood count and to separate out parasites, white cells and plasma for subsequent immunological assays and DNA extraction (see below). Thereafter an annual cross sectional survey will be conducted before each long rains at which a venous blood sample will be collected

b) At annual cross sectional surveys 50 adults in each site will have a venous sample (up to 20mls) taken for assays described below. The reason for having a sample of adults is to provide comparative values for individuals for the most immune individuals in the populations and to support assay optimization for cellular assays which typically require larger numbers of cells.

Blood sampling in cases of severe malaria detected in hospital and non severe malaria detected at OPD

A venous blood sample (up to 5mls) will be taken for preparation of blood films and separation of parasites, white cells and plasma for subsequent immunological assays and DNA extraction (see below). A convalescent venous sample (up 5 mls) will be taken three weeks after the

first sample. In a subset of 250 patients further convalescent venous samples will be collected, six, twelve and twenty four weeks for repeat immunological assays to establish the kinetics of responses.

## 8.5 Immune parameters to be measured

The aim of having a well characterised epidemiological framework is to be able to make parallel and sequential measurements of putative protective immune responses over time. Not all of the following immune parameters will be measured at all surveys, selection of which parameters to measured at a given survey will be based on a combination of volumes of plasma and cells required, having a validated assay, and level of protection shown (i.e. for immune responses found to be associated with protection, repeat studies will be performed in subsequent malaria seasons to validate the result).

### Immune responses to pre erythrocyic stages

We will use direct and cultured ELISPOT and FACS analysis of cell division and intracellular cytokine staining to analyse T cells responses to pre erythrocytic antigens thought to induce protective immune responses. We will also use Real Time PCR to detect antigen specific RNA production, encoding cytokines and other markers of cellular responses. This allows us to look in more detail at regulatory T cell responses.

### immune responses to merozoites

We will take three complementary approaches to identifying the targets of protective immune responses:

First, We will sequence genes coding for putative target antigens in parasites sampled at both cross sectional survey and from acute clinical episodes of malaria and apply molecular evolutionary and population genetic analyses that have been used to identify signatures of positive selection on protein coding sequences[17-19]. Sequencing will normally take place in Kilifi other than when the volume of samples is so high that we need access to a higher through put sequencing facility, in which case we will perform sequencing at collaborators labs in the UK.

Second, we test the ability of subjects sera to inhibit the invasion or growth of *P falciparum* in functional assays performed *in vitro* using cultured parasites and, in collaboration with colleagues at WEHI, using transgenic lines, and clonal isolates defined with respect to invasion pathway use, and antigen polymorphisms [20-23] , Methods will be optimised in James Beesons lab at WEHI and be moved to Kilifi once well established.

Third, we will analyse class and subclass specific antibody to merozoite antigens, with particular emphasis on identifying allele specific and cross-reactive responses and relating them to phenotypic variation in invasion pathways. We will examine erythrocyte binding proteins, *P. falciparum* rhoptry protein homologues (PfRH) and other merozoite antigens including MSP2, MSP1 – Block 2, MSP2 and (AMA1) and MSP3 , This work will initially be performed by standard ELISA in Kilifi but we will also explore with collaborators at WEHI a high through put system for performing antigen specific assays, with a view to transferring it to Kilifi.

## Immune responses to neo antigens on the infected red cell surface

Immune responses to surface antigens expressed on the infected red cell surface will be measured in standard agglutination and FACs assays using cultured *P falciparum* including transgenic parasites developed by our collaborators at WEHI and in functional *in vitro* assays of opsonisation and cytoadherence .

We will focus on the identification of genes encoding variant surface antigens (vsa's) with a high frequency of recognition ie common variants (vars)) that are expressed on the infected red cell surface. We will examine the kinetics and regulation of responses to these vsa's and the role and mechanisms of such responses in protecting from clinical disease.

We have recently developed an approach to the identification of common variants. Despite their diversity, all *var* genes contain a domain (DBLα) that can be amplified using generic PCR primers and sequenced, providing a "tag" for each gene [24,25]. We have established a set of putative 'ground rules' for how the sequence of the tag region ("feature strings") can predict the structure of the *var* genes to which they belong. We will investigate whether specific var feature strings are associated with common variants (as defined by previous serological techniques) and with severe disease. We will also examine the role of within host selection pressure from specific antibodies of different subclasses (by FACS analysis), and from the presence of polymorphisms in host receptors (CD36, CD31, ICAM1, CR1) for the infected red cell in controlling the expression of common variants. Having better defined a set of commonly recognised var gene products, we will examine the relationship of humoral responses to these products and protection from clinical disease. In parallel studies we will examine the T cell responses to common variants.

## Investigating the regulation of immune responses

We will extend initial observations on possible defects in B cell memory (SSC770 B cell memory and antibody memory to Plasmodium falciparum blood stage antigens: a descriptive study) by investigating in more detail the cellular basis of humoral immune responses in asymptomatic individuals and in children during and after acute malaria infection, in relation to age and exposure. We will begin by establishing age and disease state specific characteristics of B cell phenotypes and differentiation by FACS analysis of surface markers. We will establish an *in vitro* system for antigen specific B-cell differentiation into memory cells or plasma cells which incorporates the role of accessory cells (monocytes and dendritic cells) and T-cell help. Together with the phenotypic characterisation of B-cells, T cells and accessory cells, these *in vitro* assays will be used to investigate antigen specific B-cell responses longitudinally.

We will also extend our previous observations on altered dendritic cell function in children with malaria to test the hypothesis that "high affinity for CD36" (measured by *in vitro* measure of adhesion to immobilised proteins) and being "rarely" recognised by immune plasma are properties of the same PfEMP-1 variants and that parasite isolates expressing PfEMP-1 with high affinity for CD36 are more likely to evoke anti-inflammatory immune responses, which may result in mild malarial disease but only short-lived and incomplete immune responses due to modulation of dendritic cell function. We will

examine the hypothesis that the nature and degree of phenotypic changes in peripheral dendritic cell populations relates to the kinetics, maturation and longevity of putative protective humoral responses.

Finally we will examine the relationship between immune responses measured above and host genetic polymorphisms. Host DNA will be extracted from blood samples using standard techniques and polymorphisms in genes potentially controlling the immune response typed by PCR. More extensive genome wide scanning approaches to typing relevant host polymorphisms will form the subject of a separate submission to the SSC.

**Investigating Interactions between parasite virulence and immunity**

The detailed functional characterisation of parasite isolates will be analysed in relation to disease phenotypes and to molecular characterisation of key merozoite and IRBC antigens.
We will define associations between specific clinical phenotypes and cytoadherence and relate these to var phenotypes and genotypes in order to identify specific molecular motifs associated with virulence. We will focus on three areas: the role of ICAM1 binding, the role of CD36 binding and rosetting. In the case of ICAM1 and CD36 we will characterise the phenotype by a combination of static assays and binding assays under flow conditions (to be established in Kilifi) to a range of purified proteins, including a set of alanine replacement ICAM1 mutants, and to a range of cell types (full details of these assays are described in SSC protocol 723) Parasites with distinct phenotypes will be genotyped, initially using primers for the DBL alpha region of PfEMP1, and subsequently for DBL beta. We will subsequently examine the role of antibodies specific for virulence motifs in protection from severe malaria. In the case of merozoite proteins we will establish phenotyping of parasites in terms of growth rate, red cell selectivity and the use of alternative invasion pathways, and relate these to clinical phenotype and the effect of allele specific immune responses to key parasite proteins involved in invasion as outlined above.

## 9 Data Management:

All data will be archived in a network accessible database management system using MySQL. Weekly surveillance data collected using the Palm handheld devices will be transferred each week into a relevant table structure in MySQL. Relevant inpatient data will be extracted from the online FileMaker Pro system and imported into the MySQL database.

Currently in the Ngerenya cohort, outpatient data that results from a field-based referral is entered into a FoxPro based data base system. This data can be uploaded into the MySQL database and will be replaced with direct entry into appropriate MySQL table structures.

Data checking routines for all routinely collected surveillance and clinical data will be developed and ran at regular intervals in order to identify and resolve logical errors in the data.

Data generated by laboratory assays will be imported into the MySQL system from the datafiles generated by scientists looking at specific immune responses. Often such data are stored in MS-Excel workbooks (.xls files) and archived copies of these files will be retained and documented.

## 10 Time Frame/Duration of the Project:

a. Pilot study ; not applicable

b. Definitive study; The current proposal is designed to cover the four year period from 2006-2010 new sub-proposals will be submitted as appropriate.

## 11 Ethical Considerations:

i) Risks to participating individuals

No procedures are proposed that constitute a major risk. Minimal risk procedures that are proposed in the community based cohorts include finger prick blood sampling three times in one year and during febrile illness episodes, and venous blood sampling once a year thereafter. For children with clinical malaria recruited at hospital or dispensaries there will be a venous sample taken at recruitment as part of normal clinical management and the child will be asked to attend for one additional sample three weeks later, and in a subset of 250 patients a maximum of four convalescent venous blood samples will be taken over a six month period. While it is acknowledged that such sampling can cause minor pain and distress, the volumes of blood sampled do not constitute a significant risk. Blood sampling will only be conducted by trained personnel.

ii) Benefit to study participants

While the primary aim of the study is not to provide a medical service for participants, it is acknowledged that involvement in the study will result in the more prompt diagnosis and treatment of malaria, and to some extent, other illnesses. Cost-free treatment will be provided for malaria and simple childhood illnesses as recognition for the inconvenience of involvement in the study.

iii) Benefits to the community

This study forms a part of the overall the CGMR-C aims towards developing a more complete understanding of how individual children develop protective immunity to malaria. As such, it is anticipated that this study will contribute to process of developing an effective malaria vaccine that will benefit all individuals living in malaria-endemic communities. The provision of enhanced clinical surveillance by KEMRI nurses and clinical officers at dispensaries will provide a general benefit to the communities in which these studies take place.

iv) Informed consent

Subjects will only be included in the study if their parents (or the subjects themselves in the case of adults) agree to their involvement, as signified by the signing of the consent document (attached). This document will be provided in Giriama, Swahili and English. As made clear in this document, subjects will be free to leave the study at any time and for whatever reason without need for an explanation and without penalty. The informed consent forms will include

the information that samples will be stored for future investigations in Kilifi and that some samples will be exported for investigations to be carried out in collaborators laboratories.

v) Confidentiality

Clinical and sample location data will be stored on an access-restricted data base.

## 12 Expected Application of the Results:

The main application of the results obtained from this study will be in the development and assessment of malaria vaccines. Firstly, although several prototype vaccines are under development, there will be a continuing need to decide on the components of new vaccines and one approach is to identify targets that are important in naturally acquired immunity. Secondly, a major barrier in testing vaccines is the absence of good in-vitro correlates of immune status. The current study will potentially contribute to both areas.

## 13 References

1. Snow, R.W., Trape, J. F. and Marsh, K. (2001). "The past, present and future of childhood malaria mortality in Africa." Trends in Parasitology **17**(12): 593-7.

2. Marsh, K. (1992). "Malaria: a neglected disease?" Parasitology 104: 553-569.

3. Gupta, S., Snow, R.W., Donnelly, C.A., Marsh, K. and Newbold, C. (1999). "Immunity to non-cerebral severe malaria is acquired after one or two infections." Nature Medicine, **5**: (3), 340-3.

4. Marsh, K., Forster, D., Waruiru, C., Mwangi, I., Winstanley, M., Marsh, V., Newton, C., Winstanley, P., Warn, P., Peshu, N., Pasvol, G. and Snow, R.W. (1995). "Indicators of life-threatening malaria in African children." The new England Journal of Medicine., 332: 1399-1404.

5. Mwangi, T.W., Ross, A., Snow, R.W. and Marsh, K. (2004). "Defining clinical malaria under differing transmission conditions in Kilifi District, Kenya." Journal of Infectious Diseases In press.

6. Kinyanjui, S.M., Mwangi, T., Bull, P.C., Newbold, C. I. and Marsh, K. (2004). "Protection against clinical malaria by heterologous immunoglobulin G antibodies against malaria-infected erythrocyte variant surface antigens requires interaction with asymptomatic infections." Journal of Infectious Diseases **190**(9): 1527-33.

7. Polley, S.D., Mwangi, T., Kocken, C.H., Thomas, A.W., Dutta, S., Lanar, D.E., Remarque, E., Ross, A., Williams, T. N., Mwambingu, G., Lowe, B., Conway, D. J. and Marsh, K. (2004). "Human antibodies to recombinant protein constructs of Plasmodium falciparum Apical Membrane Antigen 1 (AMA1) and their associations with protection from malaria." Vaccine **23**(5): 718-28.

8. Kinyanjui, S.M., Bull, P., Newbold, C.I. and Marsh, K. (2003). "Kinetics of antibody responses to Plasmodium falciparum-infected erythrocyte variant surface antigens." Journal of Infectious Diseases **187**(4): 667-74.

9. Collins, W.E., Jefferey, G.M. (2003) "A retrospective examination of mosquito infection on humans infected with Plasmodium falciparum." American Journal of Tropical Medicine & Hygiene .68(3):366-71.

10. Day, K.P. and Marsh, K. (1991). "Naturally acquired immunity to plasmodium falciparum. " Immunology Today, 129: 68-70.

11. Anders R.F., McColl D.J. and. Coppel R.L, (1993) "Molecular variation in Plasmodium falciparum: polymorphic antigens of asexual erythrocytic stages." Acta Tropica. **53:** 239-253.

12. Kyes, S., Horrocks, P. and Newbold, C. (2001). "Antigenic variation at the infected red cell surface in malaria." Annual Review of Microbiology **55**: 673-707.

13. Marsh, K., Kinyanjui, S. (2006). "Immune effector mechanisms in malaria." Parasite Immunology 28(1-2): 51-60.

14. Nyakeriga, A.M., Troye-Blomberg, M., Chemtai, A.K., Marsh, K. and Williams, T.N. (2004). "Malaria and nutritional status in children living on the coast of Kenya." The American Journal of Clinical Nutrition **80**(6): 1604-10.

15. Snow, R.W., Omumbo, J.A., Molyneux, C.S., Obiero, J.O., Palmer, A., Weber, M.W., Pinder, M., Nahlen, B., Obonyo, C., Newbold, C., Gupta, S. and Marsh, K. (1997). "Relation between severe malaria morbidity in children and level of Plasmodium falciparum transmission in Africa." Lancet **349**: 1650-1654.

16. Blackman, M.J., Heidrich, H-G., Donachie, S., McBride, J.S. and Holder, A.A. (1990) "A single fragment of a malaria merozoite surface protein remains on the parasite during red cell invasion and is the target of invasion-inhibiting antibodies." The Journal of Experimental Medicine.;172:379-382

17. Kreitman, M., (2000) "Methods to detect selection in populations with application to the human." Annual Review of Genomics and Human Genetics **1:** 539-559.

18. Conway, D.J., Cavanagh, D.R., Tanabe, K., Roper, C., Mikes, Z.S., Sakihama, N., Bojang, K.A., Oduola, A.M., Kremsner, P.G., Arnot, D E., Greenwood, B.M. and McBride, J.S. (2000). "A principal target of human immunity to malaria identified by molecular population genetic and immunological analyses." Nature Medicine **6**(6): 689-92.

19. Polley, S.D., Chokejindachai, W. and Conway, D. J. (2003) "Allele frequency based analyses robustly identify sites under balancing selection in a malaria vaccine candidate antigen." Genetics **165:** 555-561.

20. O'Donnell, R.A., de Koning-Ward T.F., Burt R.A., Bockarie, M., Reeder, J.C., Cowman, A.F., Crabb, B.S. (2001) "Antibodies against merozoite surface protein

(MSP)-1(19) are a major component of the invasion-inhibitory response in individuals immune to malaria." The Journal of Experimental Medicine;193:1403-12

21. Duraisingh, M.T., Maier, A.G., Triglia, T. and Cowman, A.F. (2003) "Erythrocyte-binding antigen 175 mediates invasion in Plasmodium falciparum utilizing sialic acid-dependent and -independent pathways." Proceedings of the National Academy of Sciences of the United States of America;100:4796-801

22. Duraisingh, M.T., Triglia, T., Ralph, S.A., Rayner, J.C., Barnwell, J.W., McFadden, G. I. and Cowman, A.F. (2003). "Phenotypic variation of Plasmodium falciparum merozoite proteins directs receptor targeting for invasion of human erythrocytes." The EMBO Journal **22**(5): 1047-57.

23. Healer, J., Murphy, V., Hodder, A.N., Masciantonio, R., Gemmill, A.W., Anders, R. F., Cowman, A.F. and Batchelor, A. (2004). "Allelic polymorphisms in apical membrane antigen-1 are responsible for evasion of antibody-mediated inhibition in Plasmodium falciparum." Molecular Microbiology **52**(1): 159-68.

24. Taylor, H.M., Kyes, S.A. Harris, D. Kriek, N. and Newbold, C. I.. (2000). "A study of var gene transcription in vitro using universal var gene primers." Molecular and Biochemical Parasitology 105(1):13-23.

25. Taylor, H.M., Kyes, S. A. and Newbold, C.I.. (2000). "Var gene diversity in Plasmodium falciparum is generated by frequent recombination events." Molecular and Biochemical Parasitology 110(2):391-7.

26. Robinson, B.A., Welch, T.L. and Smith. J.D. (2003). "Widespread functional specialization of Plasmodium falciparum erythrocyte membrane protein 1 family members to bind CD36 analysed across a parasite genome." Molecular Microbiology 47(5):1265-78.

27. Kirchgatter, K., and Portillo Hdel A. (2002.) "Association of severe noncerebral Plasmodium falciparum malaria in Brazil with expressed PfEMP1 DBL1 alpha sequences lacking cysteine residues." Molecular Medicine 8(1):16-23.

## 14 Budget

| | US dollars | Kshs. |
|---|---|---|
| *Personnel*: Personnel, salaries and benefits disbursement. | 200,000.00 | 14,800,000.00 |
| ***Patients:*** Patients costs, travel, food and/or supplies | 4,000.00 | 296,000.00 |
| ***Supplies*** | 160,000.00 | 11,840,000.00 |
| ***Travel and accommodation:*** Local or field travel<br>International/Local conferences | 80,000.00<br>6,000.00 | 5,920,000.00<br>444,000.00 |
| ***Transport:*** Transportation, vehicle repairs, insurance etc | 80,000.00 | 5,920,000.00 |
| *Office Costs:* Operating expenses, postages, printing, etc | 10,000.00 | 740,000.00 |
| **Sub Total** | **540,000.00** | **39,960,000.00** |
| Contingency fund (15% including inflation) | 81,000.00 | 5,994,000.00 |
| **Grand Total** | **621,000.00** | **45,954,000.00** |

This proposal will be funded through the KEMRI Wellcome Trust programme and does not incur additional overheads

## 15 Appendices

A Role of each participating investigator.

| | |
|---|---|
| K Marsh | Principal Investigator, design, overall supervision |
| T Williams | Design of cohort studies, supervision of studies relating human genotype to Immune response |
| T Mwangi | Design and supervision of field studies |
| S Kinyanjui | Studies on merozoite proteins and invasion |
| B Lowe | Coordination of laboratory based studies |
| F Ndungu | Studies on regulation of cellular responses |
| P Bejon | Design of Junju cohort and studies on response to pre erythrocytic stages |
| P Bull | Studies on immune response to VSA's on infected red cell surface |
| F Osier | (PhD student) Studies on allele specific response to merozoite proteins |
| C Mugenyhi | Studies on invasion inhibiting antibodies |
| G Fegan | Statistician |
| G Warimwe | (PhD student) Studies on VSA's on infecting red cell surface |
| E Nduati | (PhD student) Studies on immune regulation |
| L Ochola | Functional characterization of infected red cells |
| B Urban, | Supervision of studies on immune regulation |
| E Ogada | Data base manager |
| M Mackinnon | study design and analysis epidemiological studies |
| D Conway | Population genetics of targets of immune response |
| J Langhorne | Collaboration on studies of immune regulation (supervisor EN) |
| J Beeson | Collaboration of studies on invasion inhibition ( supervisor CM) |
| A Farnert | Collaboration on genotyping on parasite genotyping |

- Curriculum vitae of each non-KEMRI investigator
- Informed consent advice

# Information sheet (Children in cohort studies)

## Integrated studies of the development of natural immunity to malaria in children in Kilifi district

Lead Investigator: Kevin Marsh, Director KEMRI-Wellcome Programme.

**What question is KEMRI trying to answer?**

KEMRI is an institution which learns more about diseases to find better ways of preventing and curing those diseases. An example is malaria, which causes much death and illness among Kenyan children. Children are protected against measles by the measles vaccine. Children could be protected against malaria if there was a malaria vaccine. In areas like this people become immune to the bad effects of malaria as they grow older. In KEMRI we are working to try to understand how people become immune to malaria. This information is important in designing vaccines against malaria.

**What does taking part involve?**

We want to find out why some people get more episodes of malaria and other illnesses than others. In order to do this we need to follow group of children over time to see how often they have malaria andother diseases. We also need to study how some of the components of the childs blood that fight diseases change over time. We need to follow about 500 children in this community. We plan to follow them over the next four years.

We are requesting your permission for your child to be one of these children. If you agree, the study will involve:

**1) Weekly visits to your home.** Fieldworkers living in this community will visit you and your child each week to ask if your child is well. At each visit we will take your childs temperature to see if they have a fever. If they are unwell but don't have a fever the fieldworker will return twice over the next 24 hours to recheck the temperature. If the child does have a fever they will collect a finger prick blood sample. Some of this blood will be tested immediately to see if the child has malaria. Some of it will be taken to the KEMRI laboratories for other tests which are described below. If your child does have malaria the field worker will report this by telephone to a KEMRI doctor and we will provide treatment for your child. **The fieldworkers will only be able to feed back malaria results, and they are not doctors.** If your child has another sort of problem, the fieldworker will advise you to visit the dispensary where KEMRI staff are working with the Ministry of Health staff to diagnose and treat illness.

**2) Four additional blood samples at set times in the first year , even if your child is well.** On three of these occasions the blood sample will be collected by finger prick. On one occasion a sample of 5mls (one teaspoon full) will be collected from a vein on your childs hand or arm.

3) **<u>After the first year one blood sample of 5 mls once a year for the next three years.</u>**

**Are there any risks to being in the study?**

Taking a blood sample by finger prick or by needle can be uncomfortable and can sometimes cause mild bruising. The amount of blood taken is completely safe and children are able to replace blood themselves.

**Are there any benefits to being in the study**

By having a weekly visit by the KEMRI fieldworker your child will have a regular check to see if they are unwell. If they have malaria they will receive immediate treatment and if they have other illnesses they will

be referred to the local dispensary to be seen by a nurse of clinical officer working with KEMRI In order to make sure that children with malaria or other illnesses have good access to care on days when the field worker is not visiting, KEMRI have placed extra nursing and clinical officer staff at the local dispensary. This benefits people in this community whether they are in the study or not in the study.

**What happens to the blood samples taken in the study?**

We want to find out why some people get more episodes of malaria and other illnesses than others. In the laboratory we will examine the blood to see if it contains substances or cells which recognise and protect against the malaria parasite. Some of these substances are "genetic" which means they are present from birth and some are things that develop as we grow older after we have been exposed to malaria.

Most of the tests will be done in the laboratories in Kilifi. Some of the tests cannot at present be done in Kenya and so will be done in laboratories overseas. Not all the tests will be done at the same time and so the blood will be stored in a freezer until the tests can be done. At the end of the study any blood that has not been used in tests will be stored so that further tests of the kind described above can be carried out in future.

(To be read to parents of children who are already in the cohort recruited under previous SCC protocol:

Your child has already been in this study for some time and we are now inviting you to continue in the study. We will ask you separately at the end of this form for permission to use continue to use samples already taken from your child up until this time and stored in Kilifi.

**How will my rights be protected?**

This research has been reviewed by a group scientists and doctors in Nairobi to make sure that the research is well designed. It was then reviewed by the Kenya national ethical committee. This is a committee of people from different walks of life, including lawyers and religious leaders, whose job is to make sure that the research conforms with national and international standards for safety and for protecting the rights of people who take part in the research. Any changes to the study that we want to make have to be approved first by the ethical committee. No tests or procedures other than the ones you have agreed to can be carried out. (Out of curiosity- is there a way in Swahili/Giriama to describe ethical?)

**What if I decide not to involve my child or change my mind later?**

Participation in research is completely voluntary. Nobody is obliged to take part and anyone who does agree can change their minds at any time. If you refuse to take part, or agree and change your mind later, there are no penalties what so ever, and you and your child will still be able to benefit form the improved level of service offered at the dispensary and at Kilifi hospital as a result of the research carried out by KEMRI.

If you require further information please contact Professor Marsh or any other rmember of the research team at the KEMRI centre, Kilifi. (telephone 0125 22063) You may also contact the National Ethical review Committee on 02 2722541

# Consent form

**Integrated studies of the development of natural immunity to malaria in children in Kilifi district**

Lead Investigator: Kevin Marsh, Director KEMRI-Wellcome Programme.

When you sign below it shows that you have read the information about the study and have been able to have any questions answered and that you have agreed to join the study. If you do not understand any part of the information sheet please ask questions. Participation in the study is voluntary and you have the right to change your mind and withdraw from the study at any time without giving a reason. Do not sign until you have answers to your questions.

I wish my child to take part in the study of how children develop immunity to malaria. I understand that this will involve weekly visits to my house by a KEMRI fieldworker for a period of up to four years. I understand that in the first year I will be asked permission to take a blood sample from my child on four occasions and that in subsequent years I will be asked permission to do this once a year. I understand that some of the blood samples may be exported to laboratories outside Kenya ☐

I consent to my childs blood being stored for testing in the future. ☐

(tick boxes to indicate consent, place a cross in the box to indicate lack of consent)

Childs name____________________________________

Signature (or thumb print) of parent_________________________________

Name in capitals ________________________

Date ________________

I have read the information sheet for "Integrated studies of the development of natural immunity to malaria in children in Kilifi district" to________________________ (parents name) in a language they understand.
I confirm that they give consent for_______________ (childs name) to take part in the study.
Signature of translator______________________

Name in capitals____________________
Date________________

If you require further information please contact Professor Marsh at the KEMRI centre, Kilifi. (Telephone 0415 22063) You may also contact the National Ethical review Committee on 02 2722541

# Information sheet (Adults in cohort studies)

## Integrated studies of the development of natural immunity to malaria in children in Kilifi district

Lead Investigator: Kevin Marsh, Director KEMRI-Wellcome Programme.

**What question is KEMRI trying to answer?**

KEMRI is an institution which learns more about diseases to find better ways of preventing and curing those diseases. An example is malaria, which causes much death and illness among Kenyan children. Children are protected against measles by the measles vaccine. Children could be protected against malaria if there was a malaria vaccine. In areas like this people become immune to the bad effects of malaria as they grow older. In KEMRI we are working to try to understand how people become immune to malaria. This information is important in designing vaccines against malaria.

**What does taking part involve?**

You have already agreed for your child to take part in the study. We also wish to ask you if you yourself will take part in the study. The reason we want are inviting some adults to be in the study is to compare the results with children and to help us make sure the labotatory tests are working well before we use blood from children.If you agree we will ask permission to take one blood sample by needle form a vein in your arm of 20 mls ( four teaspoons) once a year for the next four years.

**Are there any risks to being in the study?**

Taking a blood can be uncomfortable and can sometimes cause mild bruising. The amount of blood taken is completely safe.

**Are there any benefits to being in the study**

The benefits to your child of being in the study have already been explained. There will be no additional benefits to you personally from agreeing to be in the study

**What happens to the blood samples taken in the study?**

We want to find out why some people get more episodes of malaria and other illnesses than others. In the laboratory we will examine the blood to see if it contains substances or cells which recognise and protect against the malaria parasite. Some of these substances are "genetic" which means they are present from birth and some are things that develop as we grow older after we have been exposed to malaria.

Most of the tests will be done in the laboratories in Kilifi. Some of the tests cannot at present be done in Kenya and so will be done in laboratories overseas. Not all the tests will be done at the same time and so the blood will be stored in a freezer until the tests can be done. At the end of the study any blood that has not been used in tests will be stored so that further tests of the kind described above can be carried out in future. If you or your child were in a previous KEMRI study in this area we will also ask permission to do the same tests on samples of blood taken in that study and stored.

**How will my rights be protected?.**

This research has been reviewed by a group scientists and doctors in Nairobi to make sure that the research is well designed. It was then reviewed by the Kenya national ethical committee. This is a committee of people from different walks of life, including lawyers and religious leaders, whose job is to make sure that

the research conforms with national and international standards for safety and for protecting the rights of people who take part in the research. Any changes to the study that we want to make have to be approved first by the ethical committee. No tests or procedures other than the ones you have agreed to can be carried out.

**What if I decide not to be in the study, or change my mind later?**

Participation in research is completely voluntary. Nobody is obliged to take part and anyone who does agree can change their minds at any time. If you refuse to take part, or agree and later change your mind there are no penalties what so ever, and you and your child will still be able to benefit form the improved level of service offered at the dispensary and at Kilifi hospital as a result of the research carried out by KEMRI.

If you require further information please contact Professor Marsh or any other rmember of the research team at the KEMRI centre, Kilifi. (telephone 0125 22063) You may also contact the National Ethical review Committee on 02 2722541

# Consent form

**Integrated studies of the development of natural immunity to malaria in children in Kilifi district**

Lead Investigator: Kevin Marsh, Director KEMRI-Wellcome Programme.

When you sign below it shows that you have read the information about the study and have been able to have any questions answered and that you have agreed to join the study. If you do not understand any part of the information sheet please ask questions. Participation in the study is voluntary and you have the right to change your mind and withdraw from the study at any time without giving a reason. Do not sign until you have answers to your questions.

I agree to take part in the study of how children develop immunity to malaria. This I understand will involve me providing a 20 ml blood sample once a year for four years. I understand that some of the blood samples may be exported to laboratories outside Kenya. ☐

I agree to my blood being stored for research in the future. ☐

(tick boxes to indicate consent, place a cross in the box to indicate lack of consent)

Volunteers name________________________________

Signature (or thumb print) ______________________________

Name in capitals ______________________

Date _______________

I have read the information sheet for "Integrated studies of the development of natural immunity to malaria in children in Kilifi district" to_____________________ (subjects name) in a language they understand. I confirm they give consent to take part in the study.

Signature of translator____________________

Name in capitals__________________

Date______________

If you require further information please contact Professor Marsh at the KEMRI centre, Kilifi. (Telephone 0415 22063) You may also contact the National Ethical review Committee on 02 2722541

# Information sheet (Children recruited at dispensaries or hospital)

## Integrated studies of the development of natural immunity to malaria in children in Kilifi district

Lead Investigator: Kevin Marsh, Director KEMRI-Wellcome Programme.

**What question is KEMRI trying to answer?**

KEMRI is an institution which learns more about diseases to find better ways of preventing and curing those diseases. An example is malaria, which causes much death and illness among Kenyan children. Children are protected against measles by the measles vaccine. Children could be protected against malaria if there was a malaria vaccine. In areas like this people become immune to the bad effects of malaria as they grow older. In KEMRI we are working to try to understand how people become immune to malaria. This information is important in designing vaccines against malaria.

You have come to the dispensary/ hospital today because your child has an illness that may be caused by malaria. We would like to invite you to allow your child to take part in a study to help find out how people become immune to malaria.

**What does taking part involve?**
**Normally your child woyld receive some blood tests to confirm the problem and then receive treatment. These things will happen in the normal way but I addition we will ask to take an extra sample of blood for research.**

If you agree for your child to be part of the study we will:

1) take a 5mls (one teaspoon) of blood from a vein on your child's arm or hand. This will be used for tests that help the doctors decide how to treat your child. And the remainder will be used for research

2) ask permission to take a further 5 mls blood sample after your child has recovered from the illness. This will be approximately three weeks from now.

We will later invite some children to return at six, twelve and twenty four weeks from now for further blood samples. We will make it clear to you whether we are asking you to return only once or a total of four times

Whether you agree to take part or not your child will receive the normal treatment for their illness.

**Are there any risks to being in the study?**

Taking a blood sample can be uncomfortable and can sometimes cause mild bruising. The amount of blood taken is completely safe, even if your child is anaemic. If your child is anaemic they will receive treatment for this along with treatment for any other illness.

**Are there any benefits to being in the study**

We will provide the cost of bus fare for you and your child to attend for the follow up blood sample(s). At that time there will be an opportunity to see a health worker, and have appropriate tests performed, if you have any concerns that your child is not completely recovered. This research may have a long term benefit to children in this community and others in similar communities through providing information that will help in the development of better ways of preventing malaria. Otherwise there are no specific benefits.

**What happens to the blood samples taken in the study?**

We want to find out how children with malaria respond to the disease In the laboratory we will examine the blood to see if it contains substances or cells which recognise and protect against the malaria parasite. Some of these substances are "genetic" which means they are present from birth and some are things that develop as we grow older after we have been exposed to malaria.

Most of the tests will be done in the laboratories in Kilifi. Some of the tests cannot at present be done in Kenya and so will be done in laboratories overseas. Not all the tests will be done at the same time and so the blood will be stored in a freezer until the tests can be done. At the end of the study any blood that has not been used in tests will be stored so that further tests of the kind described above can be carried out in future.

**How will my rights be protected?**

This research has been reviewed by a group scientists and doctors in Nairobi to make sure that the research is well designed. It was then reviewed by the Kenya national ethical committee. This is a committee of people from different walks of life, including lawyers and religious leaders, whose job is to make sure that the research conforms with national and international standards for safety and for protecting the rights of people who take part in the research. Any changes to the study that we want to make have to be approved first by the ethical committee. No tests or procedures other than the ones you have agreed to can be carried out.

**What if I decide not to involve my child or change my mind later?**

Participation in research is completely voluntary. Nobody is obliged to take part and anyone who does agree can change their minds at any time. If you refuse to take part, or agree and alter change your mind there are no penalties what so ever, and you and your child will still be able to benefit form the improved level of service offered at the dispensary and at Kilifi hospital as a result of the research carried out by KEMRI.

If you require further information please contact Professor Marsh or any other rmember of the research team at the KEMRI centre, Kilifi. (telephone 0125 22063) You may also contact the National Ethical review Committee on 02 2722541

# Consent form

**Integrated studies of the development of natural immunity to malaria in children in Kilifi district**

Lead Investigator: Kevin Marsh, Director KEMRI-Wellcome Programme.

When you sign below it shows that you have read the information about the study and have been able to have any questions answered and that you have agreed to join the study. If you do not understand any part of the information sheet ask questions. Participation in the study is voluntary and you have the right to change your mind and withdraw from the study at any time without giving a reason. Do not sign until you have answers to your questions.

I agree to take part in the study of how children develop immunity to malaria. I understand that I will be asked permission to take a 5ml blood sample from my child during this illness and that I will be asked to return to clinic in approximately three weeks time for a second blood sample to be taken. I understand that some of the blood samples may be exported to laboratories outside Kenya. ☐

**THIS SECTION TO BE READ ONLY FOR THE SUBSET OF CHILDREN INVITED TO PARTICIPATE IN EXTENDED FOLLOW UP:**

I understand that I will then be asked permission to return to clinic on three more occasions at approximately six, twelve and twenty four weeks from now ☐

I agree to blood being stored for testing in the future. ☐
(tick boxes to indicate consent, place a cross in the box to indicate lack of consent)

Childs name______________________________

Signature (or thumb print) of parent______________________________

Name in capitals ______________________

Date _______________

I have read the information sheet for "Integrated studies of the development of natural immunity to malaria in children in Kilifi district" to____________________ (parents name) in a language they understands. I believe they give consent for_____________ (childs name) to take part in the study.
Signature of translator____________________

Name in capitals__________________
Date_______________

If you require further information please contact Professor Marsh at the KEMRI centre, Kilifi. (Telephone 0415 22063) You may also contact the National Ethical review Committee on 02 2722541

# Information sheet for children in the Kilifi Birth Cohort invited to renew participation for extended follow up

Lead Investigator: Kevin Marsh, Director KEMRI-Wellcome Programme.

**Background to the study.**

KEMRI is an institution which learns more about diseases to find better ways of preventing and curing them. Examples include malaria, pneumonia and other serious disease which cause much death and illness among Kenyan children. In KEMRI we are working to try to understand how peoples bodies learn to fight diseases. This information will be used to help to find better ways of preventing and treating diseases

Shortly after your child was born you agreed to be in a study in which we have seen your child every three months for a health check and blood sample. We first asked you permission to follow your child till they were two years old. Now that your child is two we would like to invite you to continue in a study in which we will follow your child up to the age of five years.

**What is the purpose of the study?**

By following your child when they are well we hope to understand how the way children fight diseases changes as they grow older. We are particularly interested in how children learn to fight malaria but many germs can cause a disease that presents like malaria and so it is important to study all illnesses that might be malaria. We do this by testing the substances in their blood that fight disease. Some of these substances are "genetic" which means they are present from birth and some are things that develop as we grow older after we have been exposed to different illnesses. Sometimes children develop illnesses which cause them to be admitted to hospital. We will compare the levels and types of substances in the blood of children who are admitted to hospital with those who are not. In this way we hope to learn what are the ways that some childrens bodies learn to fight disease. This information is important to help design new ways of preventing or treating diseases, such as new vaccines or medicines.

**What does taking part involve?**

If you agree for your child to be part of the study we will ask you to continue to bring your child to the KEMRI clinic for a health check every six months. At each visit we will ask questions about your child's health and examine your child. We will ask permission to take a 5ml blood sample (one teaspoon) from a vein on your child hand or arm. If your child has any health problems we will perform tests to find out what the problem is and will provide treatment at the KEMRI clinic.

If your child is ever admitted to hospital we will ask permission to take a 5 ml blood sample at that time.

**Are there any risks to being in the study?**

Taking a blood sample can be uncomfortable and can sometimes cause mild bruising. The amount of blood taken is completely safe and children are able to replace blood themselves if they are healthy.

**Are there any benefits to being in the study**

Your child will benefit from attending for a six monthly check up at which any problems will be investigated and treated. We will provide the cost of taxi (bus) fare for you and your child to attend for the regular check ups. Otherwise there are no specific benefits.

**What happens to the blood samples taken in the study?**

We want to find out how children develop the ability to fight diseases. In the laboratory we will examine the blood to see if it contains substances or cells which recognise and protect against germs that cause serious disease such as malaria, pneumonia. Some of these substances are "genetic" which means they are present from birth and some are things that develop as we grow older.

Most of the tests will be done in the laboratories in Kilifi. Some of the tests cannot at present be done in Kenya and so will be done in laboratories overseas. Not all the tests will be done at the same time and so the blood will be stored in a freezer until the tests can be done. At the end of the study any blood that has not been used in tests will be stored so that further tests of the kind described above can be carried out in future. If you or your child were in a previous KEMRI study in this area we will also ask permission to do the same tests on samples of blood taken in that study and stored.

**How will my rights be protected.**

This research has been reviewed by a group scientists and doctors in Nairobi to make sure that the research is well designed. It was then reviewed by the Kenya national ethical committee. This is a committee of people from different walks of life, including lawyers and religious leaders, whose job is to make sure that the research conforms with national and international standards for safety and for protecting the rights of people who take part in the research. Any changes to the study that we want to make have to be approved first by the ethical committee. No tests or procedures other than the ones you have agreed to can be carried out.

**What if I decide not to involve my child or change my mind later?**

Participation in research is completely voluntary. Nobody is obliged to take part and anyone who does agree can change their minds at any time. If you refuse to take part, or agree and change your mind later there are no penalties what so ever, and you and your child will still be able to benefit form the improved level of service offered at the dispensary and at Kilifi hospital as a result of the research carried out by KEMRI.

If you require further information please contact Professor Marsh or any other rmember of the research team at the KEMRI centre, Kilifi. (telephone 0125 22063) You may also contact the National Ethical review Committee on 02 2722541

# Consent form

## For Children in the Kilifi Birth Cohort invited to renew participation for extended follow up

Lead Investigator: Kevin Marsh, Director KEMRI-Wellcome Programme.

When you sign below it shows that you have read the information about the study and have been able to have any questions answered and that you have agreed to join the study. If you do not understand any part of the information sheet ask questions. Participation in the study is voluntary and you have the right to change your mind and withdraw from the study at any time without giving a reason. Do not sign until you have answers to your questions.

I agree to continue in the study of how children develop resistance to malaria and other serious diseases. I understand that I will be asked to bring my child to the KEMRI clinic every six months and that I will be asked permission to take a 5ml blood sample from my child during each visit. I understand that if my child is admitted to hospital up to the time of their fifth birthday I will be asked permission to take a 5 ml blood sample during the admission. I understand that some of the blood samples may be exported to laboratories outside Kenya. ☐

I agree to blood being stored for testing in the future. ☐

(tick boxes to indicate consent, place a cross in the box to indicate lack of consent)

Childs name___________________________________

Signature (or thumb print) of parent__________________________________

Name in capitals _________________________

Date _________________

I have read the information sheet for ""For Children in the Kilifi Birth Cohort invited to renew participation for extended follow up"

to________________________ (parents name) in a language they understands. I believe they give consent for_______________ (childs name) to take part in the study.
Signature of translator_______________________

Name in capitals____________________
Date________________

If you require further information please contact Professor Marsh at the KEMRI centre, Kilifi. (Telephone 0415 22063) You may also contact the National Ethical review Committee on 02 2722541
