# Supplementary material for: Extended Follow-Up Following a Phase 2b Randomized Trial of the Candidate Malaria Vaccines FP9 ME-TRAP and MVA ME-TRAP among Children in Kenya
Source: PLoS One. 2007 Aug 15;2(8):e707. doi: 10.1371/journal.pone.0000707 (PMC1940326; doi:10.1371/journal.pone.0000707)
Supplement: Analysis Plan S1 — Analysis Plan for second 9 months follow up (0.03 MB DOC) [file pone.0000707.s004.doc]

­­­Continued monitoring for VAC 32 (Safety, immunogenicity and efficacy against febrile malaria of the candidate vaccines FP9 ME-TRAP and MVA ME-TRAP for children in an endemic area)

Analysis Plan, Version 2. 27^th^ September 2006

P. Bejon

Children immunised under the VAC 32 protocol were initially followed up for 9 months post vaccination (from 2 weeks after the last vaccination until 1^st^ February 2006).

The children in this study underwent continued surveillance for episodes of malaria, using identical methodology as for the VAC 32 study.

Continued monitoring for a further 9 months (ending October 2006) was specified in the analysis plan for the main study. The detailed plan for this analysis is specified here.

The primary analysis was conducted at the end of the first 9 months monitoring, when the trial was unblinded. No significant efficacy was identified, and so there is no primary hypothesis under test in the subsequent 9 months monitoring. The following further analyses for extended monitoring are therefore all considered secondary. These analyses follow the same methodology and pattern as used for the first 9 months monitoring plan.

Analysis populations

Efficacy will be analysed by intention to treat (ITT); i.e. everyone randomised with follow up data (at least one visit during the second monitoring period), and then according to protocol (ATP), ie restricted to those receiving full and correct vaccinations, who were followed up at least once during the second monitoring period..

Database handling

Data is currently held in a pendragon database, complied by Edna Ogada, the database manager for ongoing cohort studies in KEMRI, Kilifi. This data will be cleaned in KEMRI before being sent to PB and PM for analysis, and a copy of the database sent to the DSMB for safekeeping at the same time.

PB and PM will then merge vaccination allocation and other demographic data from the original VAC32 database. Edna Ogada does not hold data on vaccination status.

Description

A trial profile will be produced, describing numbers of subjects undergoing continued follow up by vaccination group. This will be presented in the context of the study profile from the original VAC 32 study.

Adverse events

Solicted adverse events and general adverse events have not been monitored during the second time-period. Severe adverse events will be tabulated by vaccine group.

Immunogenicity

No further immunological studies have taken place.

Efficacy

The primary endpoint for analysis will be febrile (or “mild”) malaria; defined as fever (axilliary temperature 37.5 degrees or higher) and greater than 2500 parasites per ul (as calculated from a thick or thin blood film using the WHO method), and secondarily fever with any parasitaemia. Mixed infections will be considered for the study endpoint, single infection with non falciparum species will not be considered.

A survival analysis of time to infection will be conducted for the whole monitoring period (beginning at 2 weeks after vaccination) until the end of the second monitoring period (October 2006). Since only 26% of subjects have had an episode of malaria, the numbers at risk at the start of the second monitoring are adequate (296 subjects), and similar between the two groups (50 events vs 40 events). Beginning survival analysis at the start of the second monitoring period would replace children who had already had an episode of malaria into the analysis. These children are at higher risk of malaria *a priori* and so their risk of malaria would, in effect, be analysed twice. The intention is to examine what additional information is available in the whole cohort after prolonged monitoring.

This analysis would be conducted primarily ITT. Significance testing would primarily be by logrank, stratifying for age (in three levels; 1-2 years old, 2-5 years and 5-7 years old at time of vaccination), village (in 5 levels) and ITN (Insecticide Treated Net) use at baseline.

Further Exploratory analysis.

Secondary analyses would also be conducted also by ATP and using the secondary endpoint of any parasitaemia with fever.

Further secondary analysis would repeat the survival model, with significance testing by logrank and stratification by age and village, but beginning at the start of the second monitoring period (i.e. February 2006).

After logrank testing, a Cox proportional hazards model would be used to estimate the confidence interval, adjusting by village, age and ITN use as categorical variables. Although ITN use varied during the study, ITN use as a time-dependent covariate had similar goodness-of-fit to ITN use at baseline. ITN use at baseline only will be used in this study.

Graphical methods will be used to examine the proportional hazards assumption. A Kaplan-Meier graph will be produced showing the number at risk at selected time points. Cumulative incidence of infection by the end of surveillance will be calculated for each group. Tests for an interaction between time of monitoring and vaccine effect will be examined.

Multiple Episodes

A poisson regression model will examine multiple episodes. This will be conducted first by ITT, then ATP cohorts, and on the whole 18 months of monitoring as well as on the second monitoring period only. Analysis will be stratified by age, ITN use and village. Tests for an interaction between time of monitoring and vaccine effect will be examined.

Continued Monitoring

The cohort will be aged 3-9 years old at completion of 18 months of monitoring. The rate of malaria episodes is reduced in the older age group (HR=0.24 for 5-7 year olds compared with 1-2 year olds) and so it is unlikely that further analysis will be helpful. However, the cohort are under continued follow up as part of ongoing studies in Kilifi, and if the DSMB considers further analysis by vaccination status indicated, on the basis of the results of this analysis, a further analysis plan will be written and submitted for another year of monitoring.
